# Ex vivo and in vivo evaluation of transsphenoidal Liqoseal application to prevent cerebrospinal fluid leakage

**DOI:** 10.1007/s00701-022-05477-3

**Published:** 2023-01-09

**Authors:** Emma M. H. Slot, Nadia Colmer, Carlo Serra, David Holzmann, Luca Regli, Tristan P. C. van Doormaal

**Affiliations:** 1 Department of Neurology and Neurosurgery, University Medical Center Utrecht, Utrecht, The Netherlands; 2 Department of Translational Neuroscience, University Medical Center, Utrecht Brain Center, Utrecht University, Utrecht, The Netherlands; 3 Department of Neurosurgery, Clinical Neuroscience Center, University Hospital Zurich, Zurich, Switzerland; 4 Department of Otorhinolaryngology, Head and Neck Surgery, University Hospital Zurich, Zurich, Switzerland

**Keywords:** Cerebrospinal fluid leakage, Case report, Device, Transsphenoidal surgery

## Abstract

**Background:**

Despite improvements in closure techniques by using a vital nasoseptal flap, the use of sealing materials, and improved neurosurgical techniques, cerebrospinal fluid (CSF) leak after transsphenoidal surgery still is a clinically relevant problem. Liqoseal® (Polyganics bv, Groningen, The Netherlands) is a CE-approved bioresorbable sealant patch for use as an adjunct to standard methods of cranial dural closure to prevent CSF leakage. This study aims to evaluate the application of Liqoseal in transsphenoidal surgery ex vivo and in vivo.

**Methods:**

1. We created an ex vivo setup simulating the sphenoidal anatomy, using a fluid pump and porcine dura positioned on a conus with the anatomical dimensions of the sella to evaluate whether the burst pressure of Liqoseal applied to a bulging surface was above physiological intracranial pressure. Burst pressure was measured with a probe connected to dedicated computer software. Because of the challenging transsphenoidal environment, we tested in 4 groups with varying compression weight and time for the application of Liqoseal. 2. We subsequently describe the application of Liqoseal® in 3 patients during transsphenoidal procedures with intraoperative CSF leakage to prevent postoperative CSF leakage.

**Results:**

1. Ex vivo: The overall mean burst pressure in the transsphenoidal setup was 231 (± 103) mmHg. There was no significant difference in mean burst pressure between groups based on application weight and time (*p* = 0.227). 2. In Vivo: None of the patients had a postoperative CSF leak. No nose passage problems were observed. One patient had a postoperative meningitis and ventriculitis, most likely related to preoperative extensive CSF leakage. Postoperative imaging did not show any local infection, swelling, or other device-related adverse effects.

**Conclusions:**

We assess the use of Liqoseal® to seal a dural defect during an endoscopic transsphenoidal procedure as to be likely safe and potentially effective.

**Supplementary Information:**

The online version contains supplementary material available at 10.1007/s00701-022-05477-3.

## Introduction

Cerebrospinal fluid (CSF) leak is a frequent complication after transsphenoidal surgery (TSS), with an overall prevalence of 3.4% [7]. The prevalence of CSF leak for indications other than pituitary adenomas (i.e., craniopharyngioma, meningioma, Rathke’s cleft cysts) is 7.1%, which is similar to that found for craniotomies [7]. CSF leak is associated with various complications such as meningitis, CSF hypotension syndrome, and intracerebral hemorrhage causing increased morbidity and mortality [4, 8]. Furthermore, hospital costs for patients with CSF leak after TSS are significantly higher than for patients without [4, 11].

Despite improvements in closure techniques by using a vital nasoseptal flap (NSF), the use of sealing materials, and improved neurosurgical techniques, CSF leak after TSS still is a clinically relevant problem, for intradural and invasive lesions, such as craniopharyngiomas or tuberculum sellae meningiomas, especially. Retrospective analyses of the use of a patch sealant, TachoSil (Takeda Pharmaceuticals, Tokyo, Japan), in TSS show variable postoperative CSF leak results ranging from 0.8 to 7.8% [2, 3, 12]. For liquid sealants, Tisseel (Baxter, Deerfield, USA) and DuraSeal (Integra Lifesciences, Princeton, USA), similar results have been reported in retrospective analyses with postoperative CSF leak ranging from 1 to 12.5% [13, 14]. Pereira et al. [14] did not find a statistically significant difference in postoperative CSF leak for the use of Tisseel® or DuraSeal®. To further improve the advancement of TSS effective solutions to prevent postoperative CSF leaks are warranted.

**Fig. 1:**
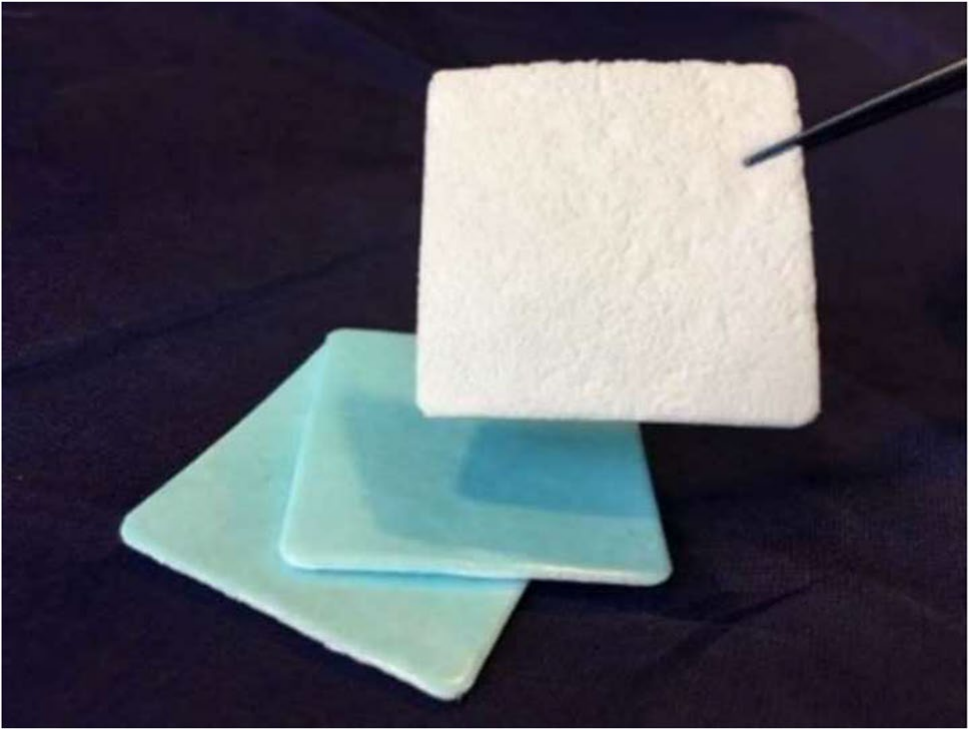
Liqoseal. Length 8 cm, width 8 cm, weight 1600 to 2000 mg. Reproduced with permission from the copyright owner [10]: Van Doormaal TPC, Germans MR, Sie M, Brouwers B, Fierstra J, Depauw PRAM, Robe PA, Regli L. Single-arm, open-label, multicenter study to evaluate the safety and performance of dura sealant patch in reducing cerebrospinal fluid leakage following elective cranial surgery: the ENCASE Trial Study Protocol. Neurosurgery. 2020 Feb 1;86(2):E203-E208. Website URL: https://journals.lww.com/neurosurgery/Fulltext/2020/02000/Single_Arm,_Open_Label,_Multicenter_Study_to.36.aspx. Neurosurgery is the official journal of the congress of neurological surgeons. The creative commons license does not apply to this content. Use of the material in any format is prohibited without written permission from the publisher, Wolters Kluwer Health, Inc. Please contact permissions@lww.com for further information

Liqoseal (Polyganics bv, Groningen, The Netherlands) is a CE (Conformité Européenne) approved bioresorbable sealant patch for use as an adjunct to standard methods of cranial dural closure. The patch is composed of a white foam layer containing Polyethylene glycol-N-hydroxysuccinimide, the adhesive component, and buffer salt [10]. The blue layer is made of polyurethane and provides the watertight seal (Fig. 1) [10]. The first in the human study (ENCASE) has shown that the patch is safe and potentially efficacious for reducing CSF leakage after intracranial surgery [10, 15].

TSS is regarded as a form of cranial surgery, and thus Liqoseal® application is not off-label [6]. However, the surrounding tissue and dimensions in this approach are different compared to a craniotomy. Therefore, this study evaluates the application of Liqoseal® in TSS in preclinical (ex vivo) settings and 3 endoscopic transsphenoidal cases.

## Methods

### Ex vivo

#### Model

**Fig. 2:**
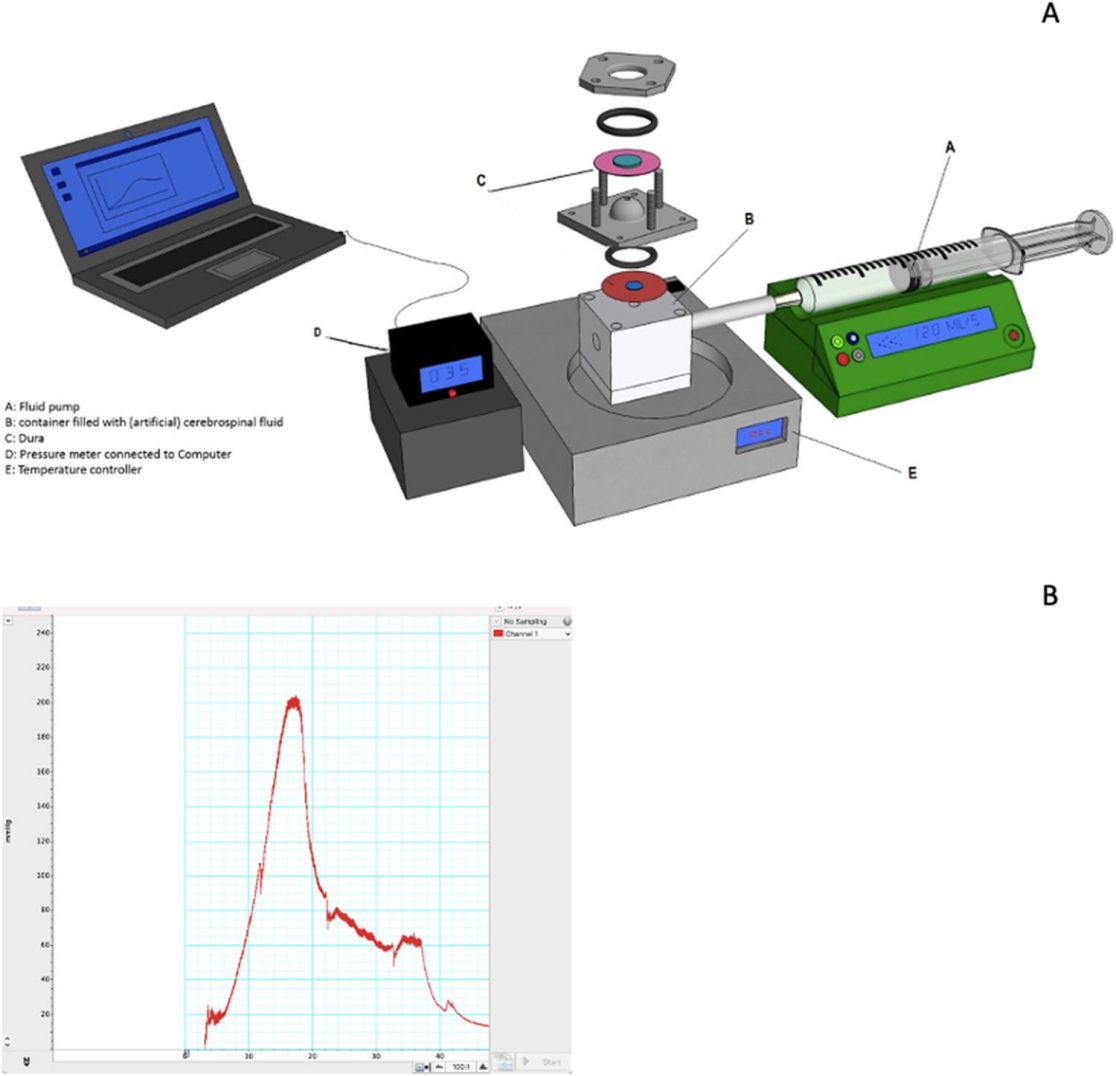
**A** Set up for burst pressure measurement. **B** Example output of burst pressure software (LabChart, AD Instruments)

We created an ex-vivo transsphenoidal burst pressure model by adapting an earlier published dural sealing model [16, 9] with a conus in the shape of the sella to mimic the application area (Fig. 2A). The dimensions of the conus (17 × 7.5 mm) were based on measurements of the pituitary gland and sella turcica on 23 anonymized MRI scans of patients with pituitary adenomas.

Cranial porcine dura was harvested at an abattoir and cut into circles with a 30 mm diameter. A circular gap of 3 mm was punched out in the center. Liqoseal® was cut into circles of 15 mm in diameter. The dura was clamped above the open pressure chamber and the Liqoseal® was applied manually to cover the gap from the outside with a 5 mm overlap.

Liqoseal® was compressed by equally and continuously applying a standardized weight on a moist gauze for a specified time period. For the cranial application of Liqoseal® a compression time of 2 min with a compression weight of 1 kg was used, to allow optimal adhesion by the formation of amide bonds between the foam layer of the patch and the dura mater [16]. However, the difficult corridor in TSS could, in practice, result in the prescribed application pressure not being met. Therefore, the acute burst pressure was evaluated with a compression weight of 1 kg and 0,25 kg. Furthermore, a shorter compression time would be clinically advantageous. Hence, we compared acute burst pressure for compression times of 2 min and 1 min, respectively.

A fluid pump with a constant flow of 2.0 mL/min of artificial CSF (EcoCyte Bioscience, Germany) was used to increase the pressure in the chamber. The pressure was continuously measured using a blood pressure probe (AD instruments MLT0670 Disposable BP transducer) connected to a computer using LabChart v8.1.14 software (ADInstruments, Australia). Burst pressure was defined as the maximum pressure in millimeters of mercury (mmHg) determined on the continuous measurement in LabChart (Fig. 2B) at the moment of fluid leakage. The aim of these experimental set-ups was to determine if Liqoseal would adhere to the dura with mean burst pressure above the higher end of the physiological intracranial pressure range (> 30 mmHg) [1] on a surface resembling the shape of the sella with varying compression weight and time during application.

#### Statistics

The required sample size to detect statistically significant differences between groups with an alpha of 0.05 and power of 90% was determined at 23 measurements per subgroup, using the power analysis for One-way Analysis of Variance (ANOVA). Input for sample size calculation was based on the results of the previous cranial and spinal measurements [16]. A total of 3 additional measurements were planned per subgroup to allow for loss of measurements due to experimental failure, so in total 104 measurements were performed. The four groups varying in compression weight (1 kg vs. 0.25 kg) and time (1 min vs. 2 min) were compared using ANOVA. Post hoc Bonferroni correction was applied to adjust for multiple comparisons. Spearman’s rank-order correlation was used to evaluate the association between burst pressure and the interval between measurement and harvesting of the dura. All analyses were performed in SPSS version 27 (IBM).

### In vivo

We performed a retrospective evaluation of all transsphenoidal surgeries in which Liqoseal® was used in the University Hospital of Zurich, Switzerland, between the 3rd of January 2020 (when Liqoseal® was approved) and the 1st of March 2022. Three Liqoseal applications were performed in these procedures. Liqoseal® was applied on the outside of the defect in all cases. All 3 patients provided a general informed consent for the use of all clinical data and imaging for research.

## Results

### Ex vivo

**Fig. 3:**
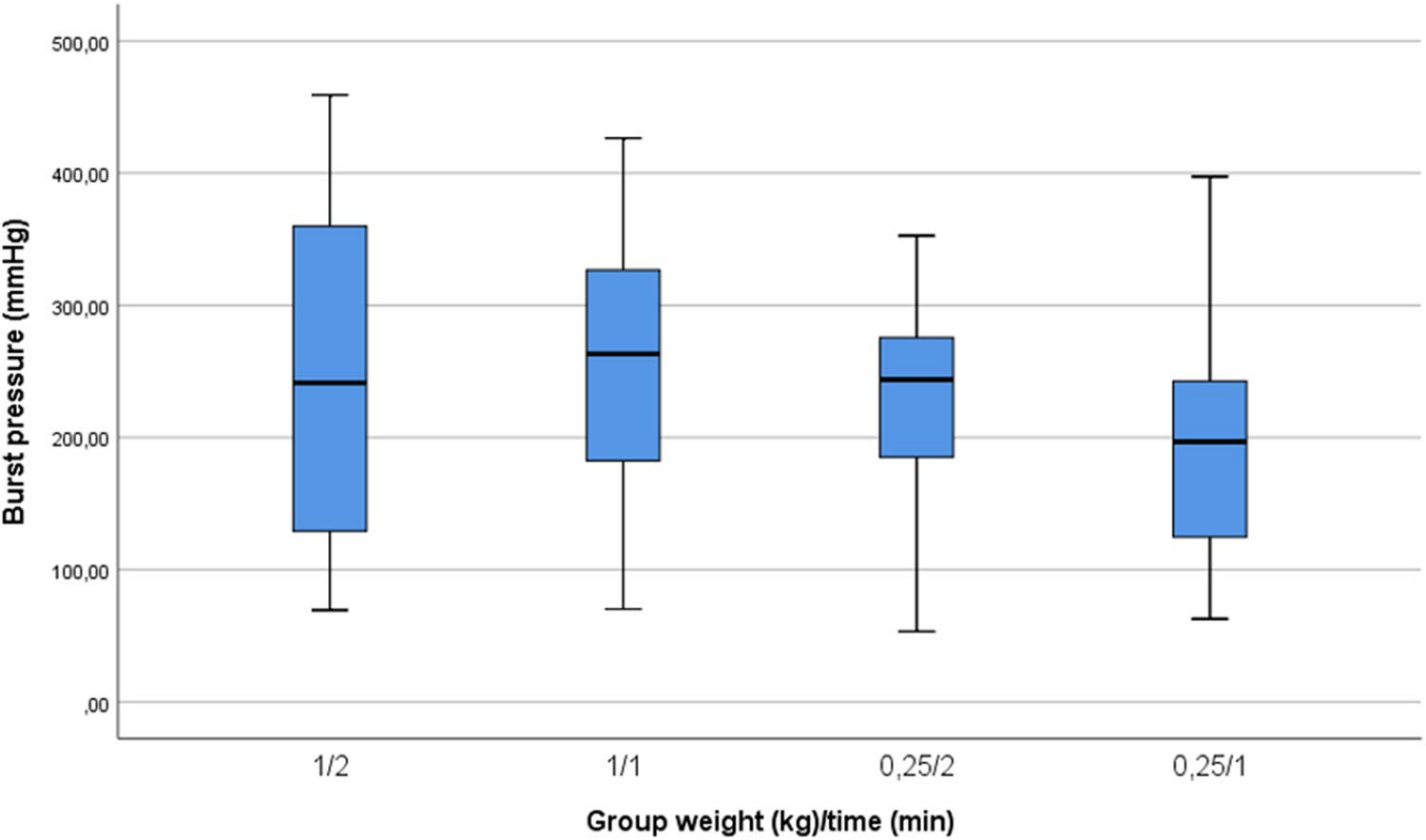
Boxplot (minimum, Q1, median, Q3, and maximum) of burst pressure in 4 groups varying compression weight and time: 1 kg/2 min, 1 kg/1 min, 0.25 kg/2 min, and 0.25 kg/1 min

A total of 100 measurements were included in the analysis. Four measurements were excluded from the analysis because leakage in the experimental setup prevented adequate pressure built-up. The overall mean burst pressure in the transsphenoidal setup was 231 (± 103) mmHg (Fig. 3, Table 1). There was no significant difference in mean burst pressure between groups based on application time and weight (*p* = 0.227).

**Table 1:** Burst pressure in 4 groups; 1 kg/2 min, 1 kg/1 min, 0.25 kg/2 min, and 0.25 kg/1mi

Group	Mean Burst Pressure (mmHg)	SD	Lowest value	Highest value	*N* included	*N* performed
1 kg, 2 min	241.4	135.0	69.4	459.0	25	26
1 kg, 1 min	257.3	102.0	70.1	426.4	24	26
0.25 kg, 2 min	229.5	77.5	53.4	352.6	25	26
0.25 kg, 1 min	199.0	85.1	62.9	397.4	26	26

*SD*, standard deviation; *N*, number

Spearman’s rank-order correlation showed no significant correlation between mean burst pressure and the interval between experiment and harvesting (*r*_*s*_ = 0.031, *p* = 0.759).

### In Vivo

Liqoseal® was applied in 3 endoscopic transsphenoidal surgeries until March 1, 2022.

#### Case 1

**Fig. 4:**
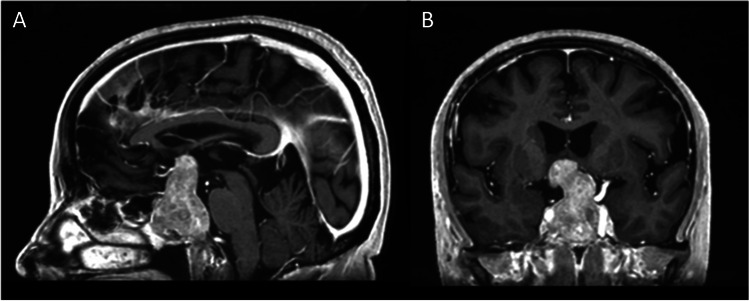
Preoperative MRI patient 1 showing a macroadenoma in **A** sagittal view and **B** coronal view

**Fig. 5:**
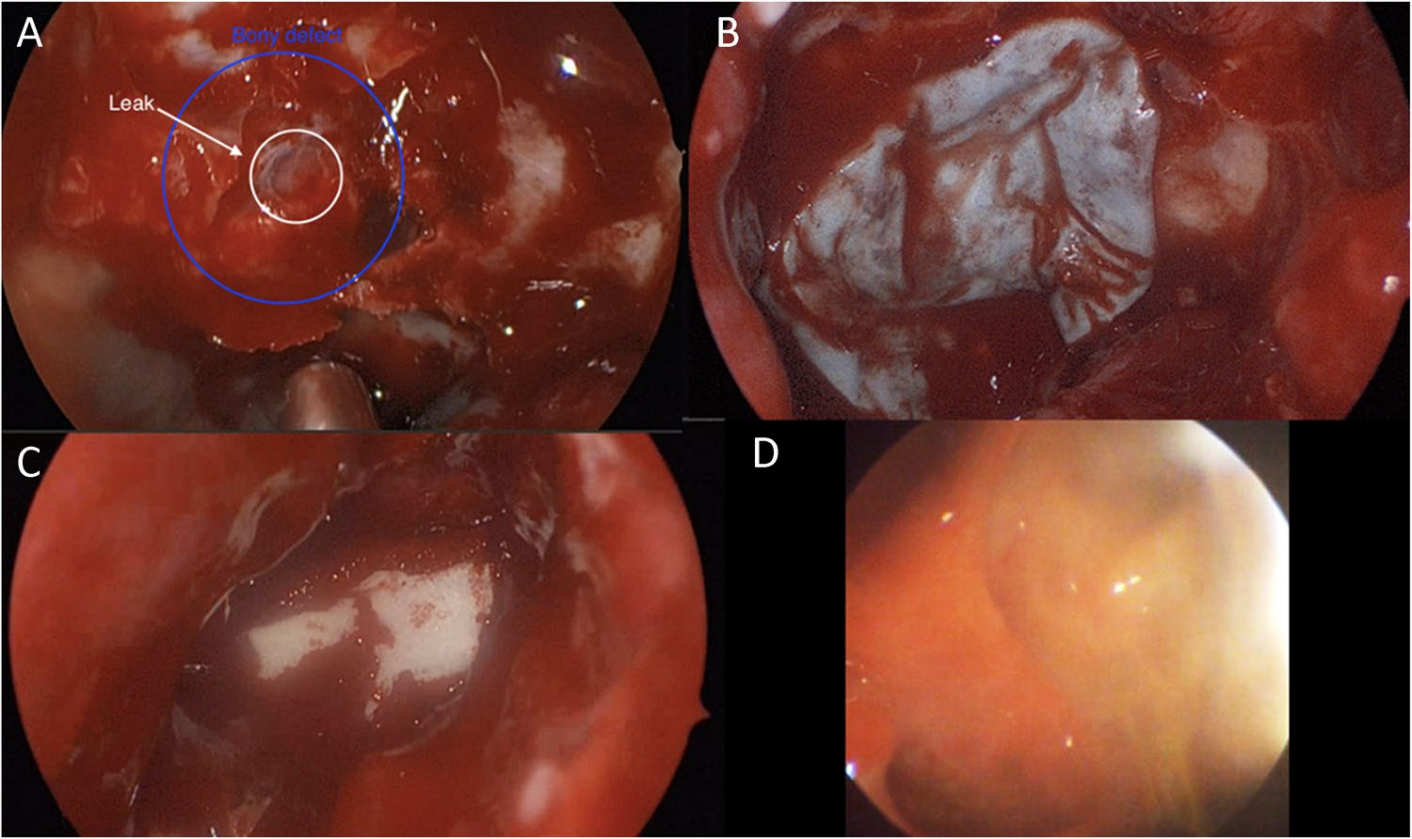
Endoscopic images of patient 1 showing **A** intraoperative CSF leakage, **B** final Liqoseal positioning, **C** intraoperative end situation, and **D** 3-month follow-up with full re-endothelisation in patient 1

**Fig. 6:**
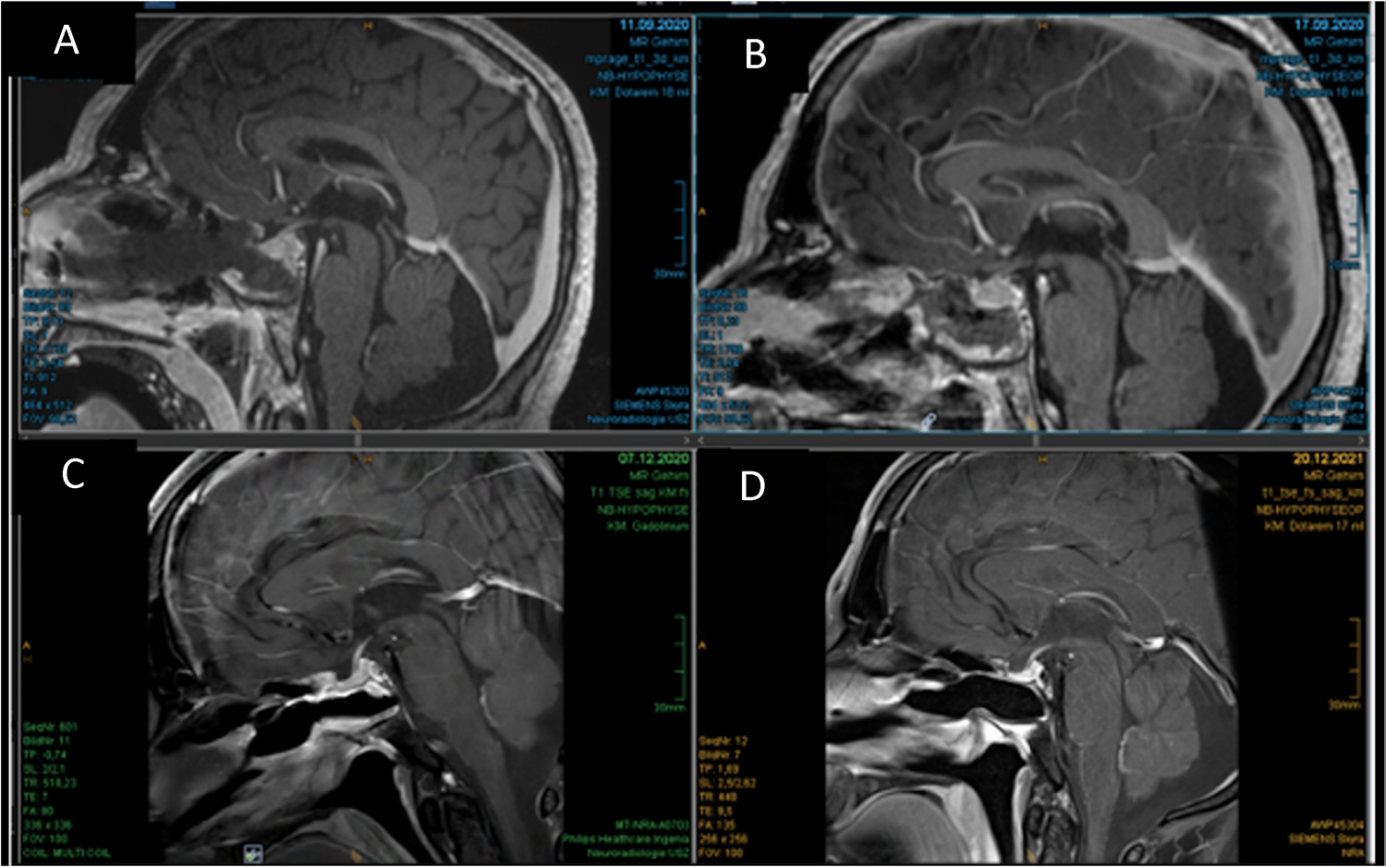
MRI follow-up patient 1 showing smoothening of the sellar wall over time. No signs of infection, swelling, or other pathological reactions were observed. **A** Intraoperative MRI (no Liqoseal), **B** day 6 postoperatively, **C** 3 months postoperatively, and **D** 15 months postoperatively

Patient 1 (63 years old male) was diagnosed with a hormone-inactive growing gonadotrophic macroadenoma (Fig. 4, Table 2, Supplementary Information 1). Intraoperatively an evident CSF leak occurred (Fig. 5A). The patient was operated using the mononostril “chopstick” approach with the aim to preserve healthy mucosal tissue [5]. Considering the small size of the defect, preparing an NSF resulting in damage to the nasal mucosa was not considered favorable. Therefore, it was decided to seal with Liqoseal® combined with external lumbar drainage (ELD). A piece of plastic was used to assess the size of the bony defect in the sella. A circular piece of Liqoseal® was cut with 10 mm margin at all sides. After trying several folding options, the piece was folded in 2 with the white side out and parachuted in holding the patch at the front tip to pull the patch forward instead of pushing it. After positioning, a series of small cottonoids was positioned over the Liqoseal® before compressing for 2 min with a 90-degree ring curette. This led to a good adherence over bone and sella region. However, a small bottom part of the sealant was hampered by loose mucosa. The Liqoseal® could be removed with a gentle pulling force via the forceps. The basal bone was cleaned, mucosa removed and a second circular piece of the same patch of Liqoseal® was applied that covered the whole sella defect with a margin of 10 mm (Fig. 5B). Positive end-expiratory pressure (PEEP) test was performed (20 cm H_2_O for 20 s) showing no leakage. The patch was covered with Tisseel® and to prevent the patch from being exposed to air and Spongostan (Ethicon, Raritan, USA) to further cover the patch and mucosa, to fill up the cavity and provide additional tissue support (Fig. 5C). A nasal packing was put in place to further provide support to the surrounding tissues and to tamponade any small bleeding afterwards. Postoperatively, no rhinoliquorrhea was observed. The ELD was removed at day 6. Patient was discharged day 8 after surgery without complications. Three-month endoscopic control showed complete re-endothelialization (Fig. 5D). At further MRI follow-up (Fig. 6) individual patch recognition was not possible, but no signs of infection or swelling of the patch were observed. During the entire follow-up period of 15 months, there were no nasal complaints and good olfactory function.

**Table 2:** Overview of cases

	Age	Sex	BMI	Smoking	Relevant medical history	Indication	Intraoperative complications	Other closure techniques	External CSF drainage	Postoperative complications	Discharge (d)	Postoperative treatment	Neurological deficit	Final follow-up (m)
1	63	M	26	Former (20 PY)	None	Macro adenoma	CSF leakage	Tisseel, Spongostan, Fat, Nose tampon	ELD day 0–6	None	7	None	None	15
2	54	F	23	No	2 times TSS	Revision CSF leakage	None	Tisseel, Spongostan, Fat, Nose tampon	ELD day 0–4 EVD day 4–40 VPS at day 40	Meningitis and ventriculitis, Hydrocephalus	44	None	Bitemporal hemianopsia	6
3	7	F	15	No	None	Clival chordoma	CSF leakage	Tisseel, Spongostan, Fat	ELD day 0–8	None	12	Proton beam therapy	Abducens nerve palsy	7

*d*, days; *m*, months; *BMI*, body mass index; *CSF*, cerebrospinal fluid; *ELD*, eternal lumbar drain; *EVD*, external ventricular drain; *VPS*, ventriculoperitoneal shunt

#### Case 2

**Fig. 7:**
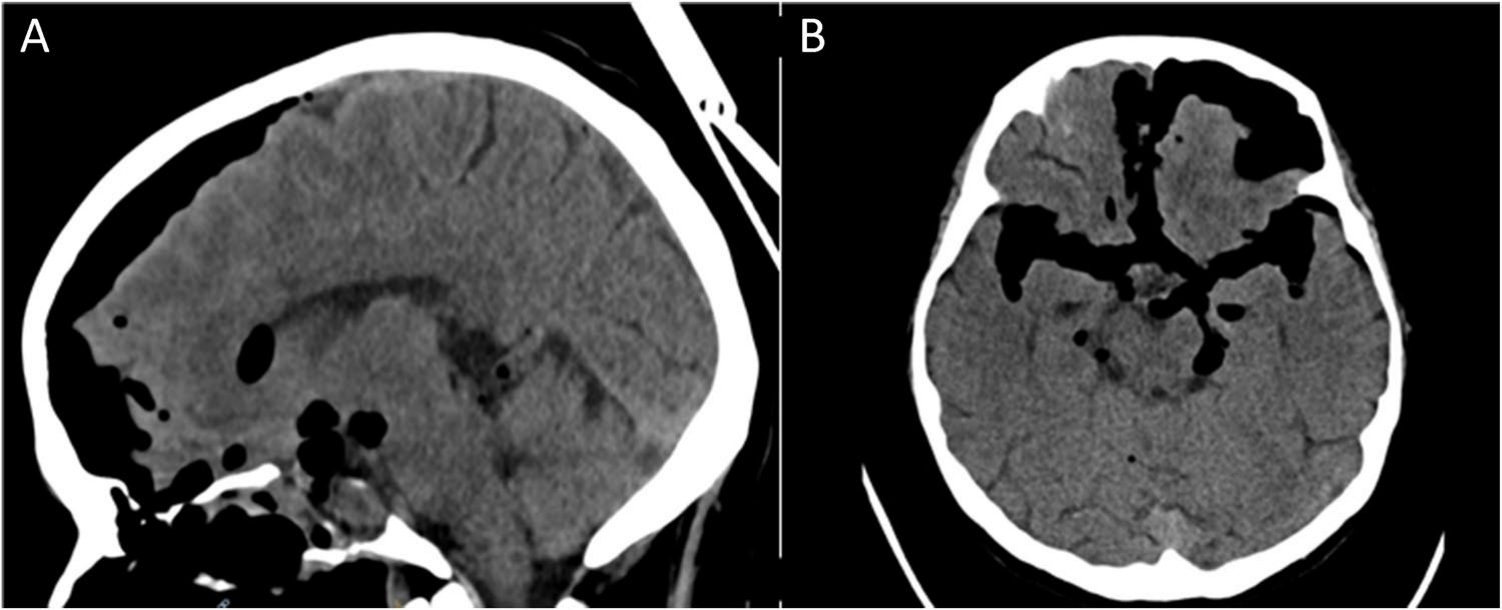
Preoperative CT patient 2 showing pneumocephalus due to CSF leakage after previous surgery in **A** sagittal view and **B** axial view

**Fig. 8:**
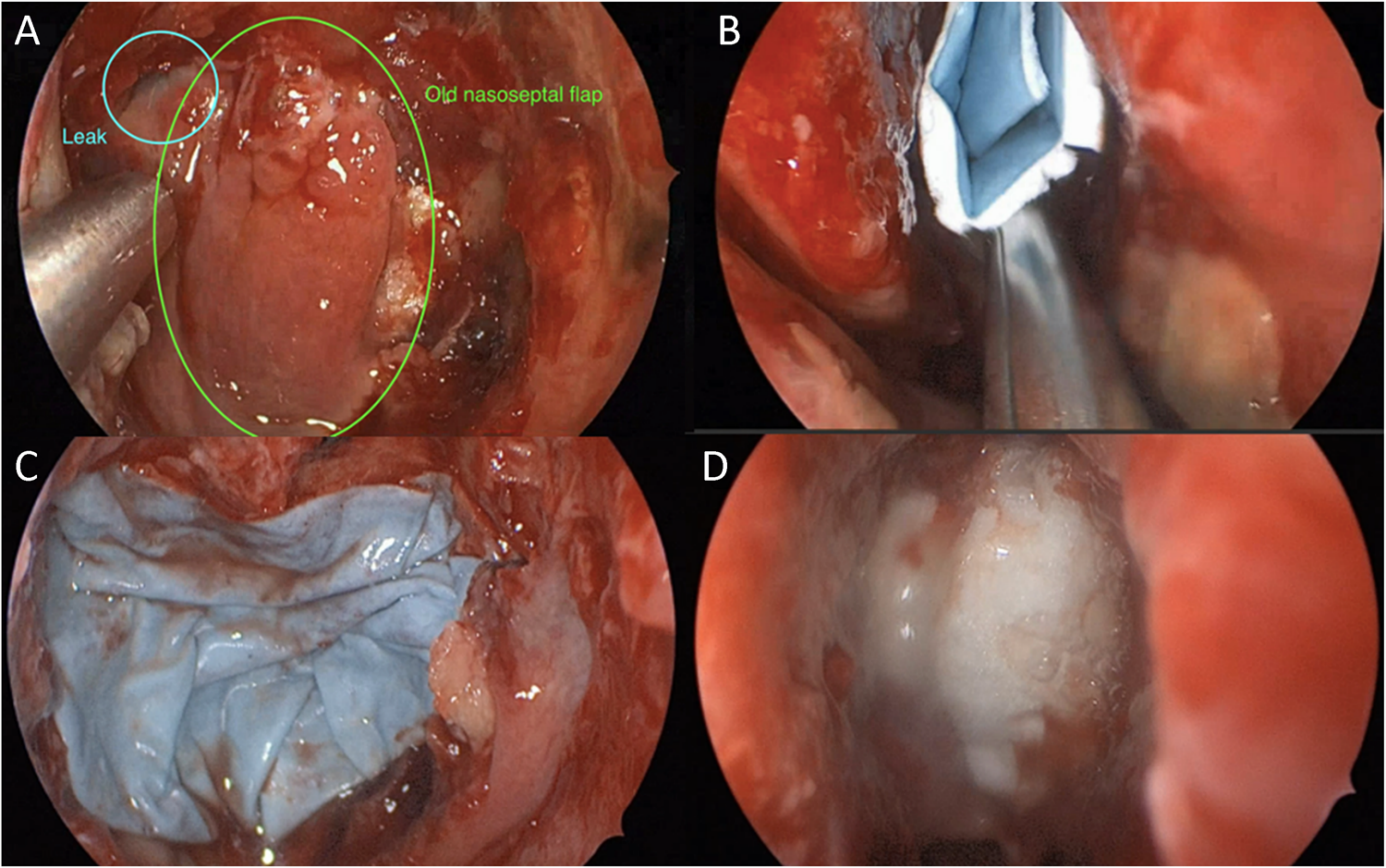
Endoscopic images patient 2 showing **A** intraoperative CSF leakage, **B** folding of Liqoseal during application, **C** final Liqoseal positioning, and **D** intraoperative end situation in patient 2

**Fig. 9:**
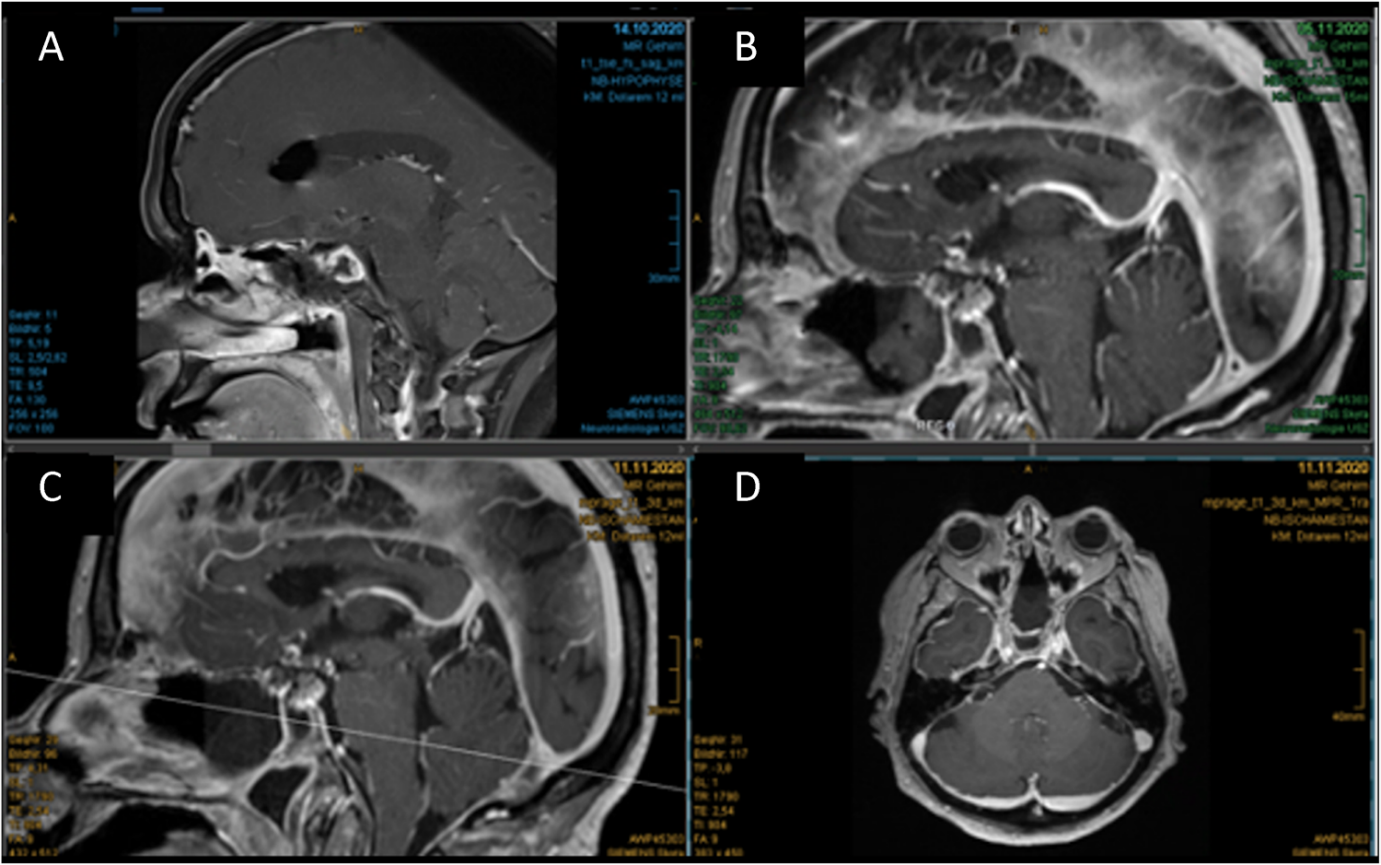
MRI follow-up patient 2 **A** 16 days before 3rd surgery, **B** day 6 after 3rd surgery, **C** day 12 postoperatively (sagittal), and **D** day 12 postoperatively (transversal) showing no swelling of the patch or signs of infection

Patient 2 (54 years old female) was diagnosed with a giant macroadenoma causing bitemporal hemianopsia (Fig. 7, Table 2, Supplementary Information 1). First surgery (day -17) was complicated by postoperative rhinorliquorrhea. A revision surgery was performed using a vascularized NSF to seal the defect and decreasing CSF pressure with ELD (day -7). The leakage continued postoperatively despite increasing CSF drainage volume. During the second revision surgery (day 0) a defect just above the vital NSF was observed (Fig. 8A). As salvage treatment a fat plug was placed in the small defect. Subsequently, Liqoseal was inserted with the same method as described in patient 1 (Fig. 8B–C). PEEP test (20 cm H_2_O for 20 s) showed no intraoperative leakage. The patch was covered with Tisseel® and Spongostan®. A nasal packing was put in place. No rhinoliquorrhea was observed after this surgery. Patient developed a combined meningitis and ventriculitis at day 4 after the 3rd surgery, which was treated with intravenous antibiotics. The ELD was exchanged for an external ventricular drain (EVD) at this day to treat the infection and resulting hydrocephalus. The treating neurosurgeon did not consider Liqoseal® as the source of the infection, hence the nose was not surgically revised. At day 12 an MRI was made (Fig. 9). Individual Liqoseal® patch recognition was not possible and there were no signs of infection or swelling of the patch. Temporary closure of the EVD resulted repeatedly in hydrocephalus (still without leakage). Therefore, a ventriculoperitoneal shunt was placed at day 40. Patient was discharged at day 44. Final follow-up was 6 months after the surgery in which Liqoseal® was applied. Visual disturbances persisted. Patient-reported no nasal complaints and good olfactory function. She refused further follow-up.

#### Case 3

**Fig. 10:**
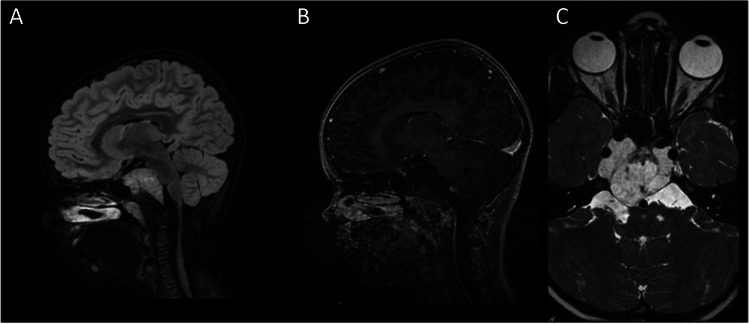
Preoperative MRI showing a clivus chordoma in **A** and **B** sagittal view and **C** axial view

**Fig. 11:**
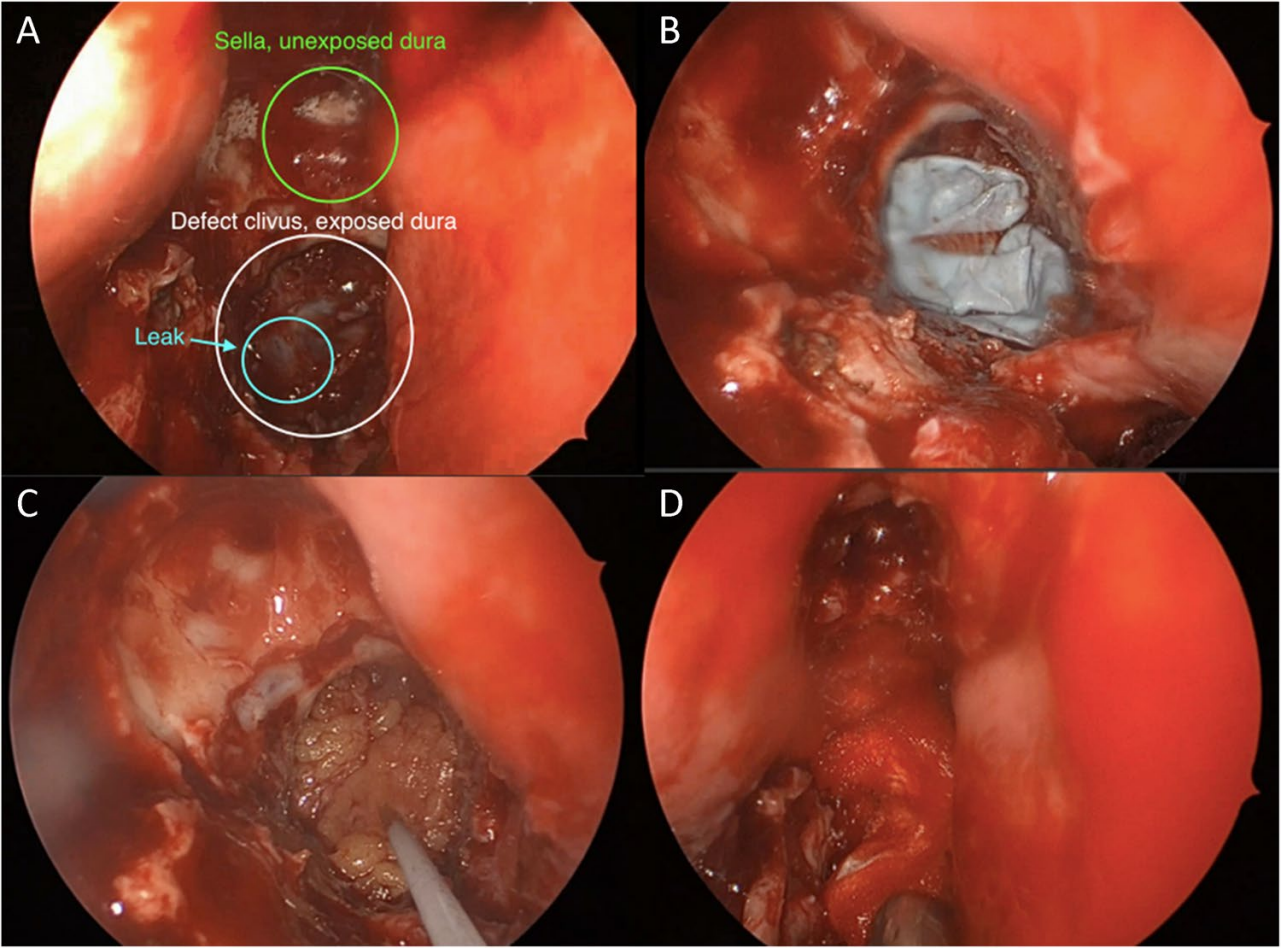
Endoscopic images showing patient 3 **A** intraoperative CSF leakage, **B** final Liqoseal positioning, **C** fat plug fixated with Tisseel on top of Liqoseal, and **D** intraoperative end situation in patient 3

**Fig. 12:**
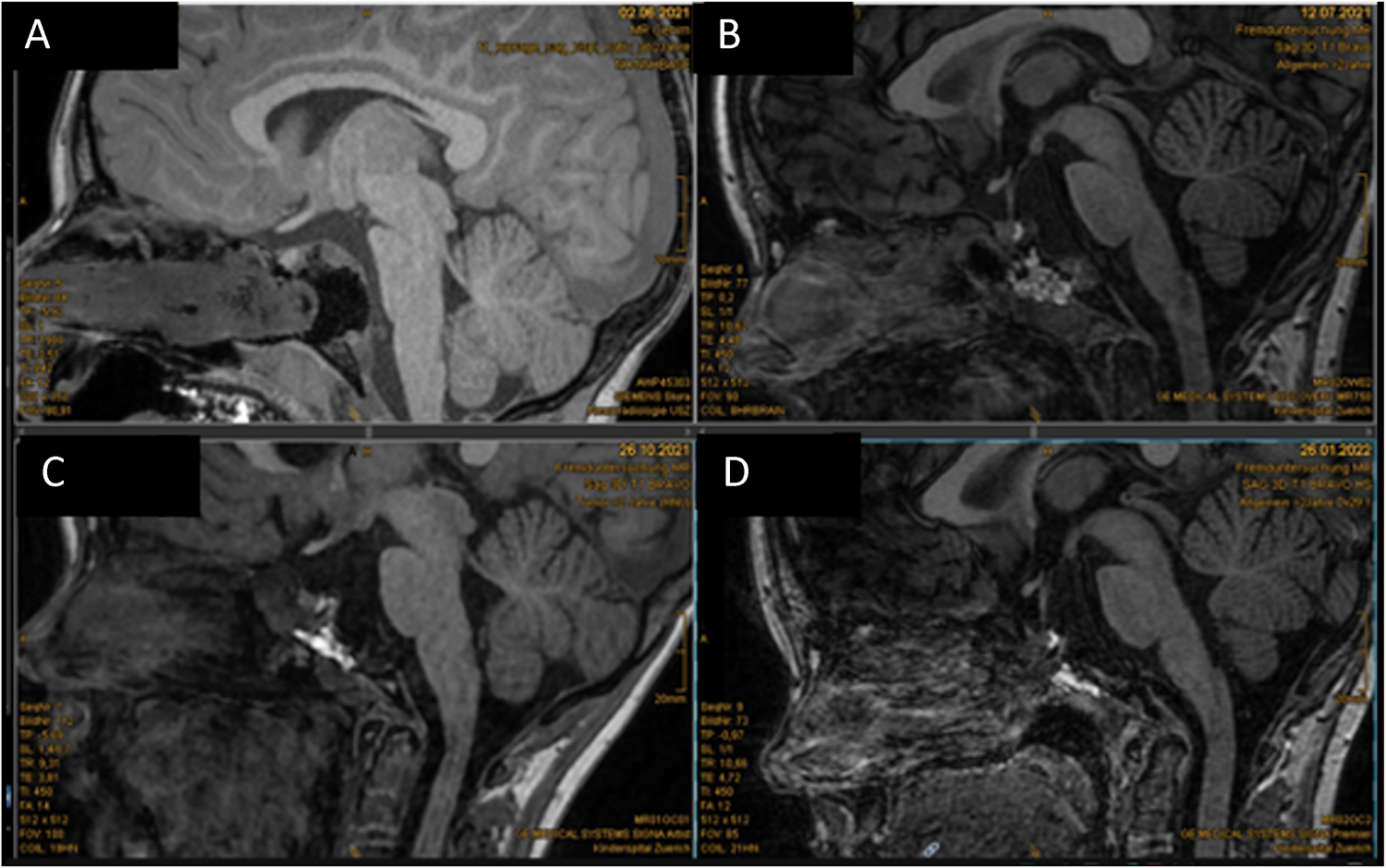
MRI results in patient 3 **A** intraoperatively, **B** 1 month postoperatively. **C** 5 months postoperatively, and **D** 8 months postoperatively, showing no swelling of the patch or signs of infection. Slow resolving of fat patch can be observed

Patiënt 3 (7 years old female) presented with an abducens nerve palsy caused by a clivus chordoma (Fig. 10, Table 2, Supplementary Information 1). After resection a large defect in the clivus resulted with a central dural defect (Fig. 11A). A NSF was not prepared and it was considered by the operating surgeon that it would be difficult in this case to make it large enough to cover the total defect appropriately. However, no dural sealants have been CE approved for use in children. So on the discretion of the operating surgeons Liqoseal® was chosen to be used off-label. This application area was deeper and flatter than in the previous 2 patients. This caused the Liqoseal® application to be more difficult and a re-application was necessary. The final positioning showed wrinkles and internal Liqoseal® folds (Fig. 11B). The operating surgeon however decided to leave the patch in place because the dural defect was covered. The Liqoseal® was covered with a fat plug harvested from the periumbilical region (Fig. 11C). Tisseel® and fat were thereafter alternately applied. Finally, the construct was covered with Spongostan® to further fill the cavity and deliver additional tissue support (Fig. 11D). No PEEP test was performed. Because of the high risk of postoperative leakage associated with the dural defect an ELD was placed intraoperatively as well. Postoperatively, no rhinoliquorrhea was observed. The ELD was removed at day 8. No postoperative complications occurred and patient was discharged at day 12 after surgery. Intraoperative and postoperative MRI showed a small chordoma rest at the cavernous sinus which was considered inoperable. The patient was radiated with proton beam 7 weeks after surgery. Latest follow-up was at 7 months after surgery. The abducens paresis persisted. Patient showed good nasal passage and olfactory function up until this time. MRI control at this timepoint showed no swelling of the Liqoseal® patch and slow resolving of the fat plug (Fig. 12).

## Discussion

This is the first study that evaluates the application of Liqoseal® during TSS. We report excellent ex vivo and in vivo results. The overall mean burst pressure of Liqoseal® in this transsphenoidal model (231 ± 103 mmHg) and mean burst pressures in individual groups based on compression weight and time were all well above physiological intracranial pressure [1]. Mean burst pressure in this model was shown to be similar to those found in our cranial and spinal model (145 ± 39 mmHg and 233 ± 81 mmHg, respectively) [16].

Liqoseal® was successfully applied during endoscopic endonasal surgery in 3 patients. Given their clinical history, each of these patients can be considered as high risk for postoperative CSF leakage. None of these patients’ postoperative CSF leakage required revision surgery or had nasal passage problems. There was one infectious complication in patient 2 that occurred 4 days after the implantation of the device. This patient was at increased risk for infection because of continuous CSF leakage prior to the surgery in which Liqoseal® was applied, and the infection was treatable with antibiotics [4]. We deem the infection unlikely to be device-related. We found no indications of safety issues for the transsphenoidal application of Liqoseal® based on these 3 patients.

### Limitations

The most important limitation of the current study is the small number of TSS cases in which Liqoseal® has been applied which does not allow for any conclusions about efficacy. Moreover, all patients in this study received an intraoperative ELD to decrease the CSF pressure and support the healing of the dura which may have positively contributed to the prevention of CSF leakage and the functioning of the patch. In addition, fibrin glue (Tisseel®) and gelatin sponge (Spongostan®) were used as a coverage. Furthermore, endoscopic inspection of the nasal mucosa (not standard of care) was performed in one patient only, showing re-endothelization.

Finally, the experimental model was designed based on the sella region. This is representative for the majority of transsphenoidal cases, but not all of them. For example, patient 3 had a clivus tumor that grew under the sella and the surface of this region does not resemble the surface of the ex vivo model. Furthermore, the gap size in the dura in the experimental setup was 3 mm in diameter. In clinical practice, the gap size in the dura, especially in cases leading to CSF leak postoperatively, may in fact be larger.

### Recommendations

Based on our experience in these first 3 cases, we think that there are a number of technical aspects to take into consideration when applying Liqoseal® in TSS. Firstly, we recommend patch sizing to allow for margins of minimally 5 mm, taking into consideration that a larger sized patch is more difficult to introduce. When fat tissue is placed under Liqoseal®, we recommend a margin of 10 mm as Liqoseal® does not adhere to fat. Secondly, we recommend to fold the patch with the white side (PEG-NHS side) outwards. This has the advantage of easier unfolding, yet does expose the foam layer to possible absorption of blood and damage. Thirdly, the patch should be held at the most distal point with a small rongeur while being introduced in the nose to exert a pulling force on the patch instead of a pushing force. Fourthly, in these 3 cases, compression for 2 min using moistened cottonoids and a patty was performed with a 90-degree bended ring curette. Despite the results of the ex vivo experiments showing that 1 min compression appears to be sufficient, we still recommend to compress for a minimum of 2 min as stated in the instructions for use for security and consistency reasons. Finally, the dural defects in the cases presented in this article were relatively small. Liqoseal® is intended for use on defects with a maximum size of 3 mm. Use over larger defects is thus off-label. We recommend to use Liqoseal® in cases with larger defects with caution and only in combination with a construct allowing endothelization and formation of new dura (i.e., covering the mucosal tissue with muscle tissue or fat). It is important to note that Liqoseal does not adhere to fat tissue and that fat tissue will resorb over time. Considering the relatively fast endothelization we have observed, the primary goal of using Liqoseal® in such cases is to overcome the time until endothelization without CSF leakage.

## Conclusion

The results of this study combined with the outcomes of the ENCASE trial [10, 15] and previous preclinical studies with regard to CSF leakage [7, 16, 9, 17–19] indicate that the use of Liqoseal® in the sphenoid sinus to seal a dural defect in TSS is likely safe and potentially effective.

## Supplementary Information

Below is the link to the electronic supplementary material.

Supplementary file1 (DOCX 18 KB) Supplementary Information 1. Additional data presentation with regard to the cases.

Supplementary file2 (MP4 376654 KB) Video part 1. Summarized videography of the first application in case 1.

Supplementary file3 (MP4 474575 KB) Video part 2. Summarized videography of the second application in case 1.

Supplementary file4 (MP4 391765 KB) Video part 3. Summarized videography of case 2.

Supplementary file5 (MP4 476432 KB) Video part 4. Summarized videography of case 3.

## Data Availability

Data is available from the corresponding author upon reasonable request.

## Abbreviations

ANOVA: Analysis of variance
CE: Conformité Européenne
CSF: Cerebrospinal fluid
ELD: External lumbar drain
EVD: External ventricular drain
MRI: Magnetic resonance imaging
mm: Millimeters
cm: Centimeters
mmHg: Millimeters of mercury
N: Number
NSF: Nasoseptal flap
PEEP: Positive end-expiratory pressure
TSS: Transsphenoidal surgery
USA: United States of America
