# Supplementary material for: Ex vivo and in vivo evaluation of transsphenoidal Liqoseal application to prevent cerebrospinal fluid leakage
Source: Acta Neurochir (Wien). 2023 Jan 9;165(6):1511–21. doi: 10.1007/s00701-022-05477-3 (PMC10227155; doi:10.1007/s00701-022-05477-3)
Supplement: Supplementary file 1 — Supplementary file1 (DOCX 18 KB) Supplementary Information 1. Additional data presentation with regard to the cases. [file 701_2022_5477_MOESM1_ESM.docx]

**Supplementary Information 1**

**Supplementary Table 1** Sealing specific variables

| **Case Nr.** | **LIQOSEAL** | **PEEP test performed** | **Other sealants** | **Other closure techniques** | **Sealing re-application** | **Long term nose problems/ sealing complications** |
| --- | --- | --- | --- | --- | --- | --- |
| 1 | DS01-024/08  Max 2021-08-19  Dur202002511 | Yes (20 cm H_2_O | Tisseel, Spongostan | Nasal packing | 1 re-application | None (15 months) |
| 2 | DS01-024/08  Max: not noted  Dur2020091111 | Yes (20 cm H_2_O) | Tisseel, Spongostan | Fat, nasal packing | None | None (6 months) |
| 3 | DS01-024/08  Max 2023-02-12  Dur2020021111 | No | Tisseel, Spongostan | Fat | 1 re-application | None (7 months) |

Nr. = number

PEEP = positive end-expiratory pressure

**Supplementary Table 2** MRI information

| **Case Nr.** | **MRI 1** | **MRI 2** | **MRI max** |
| --- | --- | --- | --- |
| 1 | Intraoperative | Day 6 | 15 months |
| 2 | Day 6 |  | Day 12 |
| 3 | Intraoperative | 4 Months | 7 months and 3 weeks |

MRI: magnetic resonance imaging

Nr. = number

**Supplementary Table 3** Body Temperature (°Celsius)

| **Case**  **Nr.** | **Day 0 (= surgery)** | **Day 1** | **Day 2** | **Day 3** | **Day 4** | **Day 5** | **Day 6** | **Day 7** |
| --- | --- | --- | --- | --- | --- | --- | --- | --- |
| 1. | 36.7 | 37.1 | 37.3 | 37.1 | 36.8 | 37.2 | 37.4 | 37.1 |
| 2. | 36.8 | 37.1 | 37.3 | - | 39.6 | - | - | - |
| 3. | 37.2 | 37.3 | 37.5 | - | 37.4 | - | - | 36.5 |

Nr. = number

**Supplementary Table 4** C-reactive Protein (mg/L)

| **Case Nr.** | **Day 0 (= surgery)** | **Day 1** | **Day 2** | **Day 3** | **Day 4** | **Day 5** | **Day 6** | **Day 7** | **Day 30** | **Day 90** | **Max FU** |
| --- | --- | --- | --- | --- | --- | --- | --- | --- | --- | --- | --- |
| 1 | 0.5 | 27 | - | 10 | - | - | - | - | - | - | - |
| 2 |  | 37 | 19 | 18 | 181 | 345 | 222 | - | 3 | - | - |
| 3 |  | 9 | - | 7 | - | - | - | - | - | - | - |

FU= follow-up

Nr. = number
